# Ultrasound guided versus blinded injection in trigger finger treatment: a prospective controlled study

**DOI:** 10.1186/s13018-023-03950-y

**Published:** 2023-06-26

**Authors:** Mahmut Tunçez, Kaya Turan, Özgür Doğan Aydın, Hülya Çetin Tunçez

**Affiliations:** 1 Department of Orthopedics and Traumatology, Izmir Katip Celebi University Ataturk Training and Research Hospital, İzmir, Turkey; 2 Department of Orthopedics and Traumatology, Istinye University, İstanbul, Turkey; 3 Department of Radiology, Izmir Bozyaka Education and Research Hospital, University of Health Sciences, İzmir, Turkey

**Keywords:** Trigger finger, Steroid injection, Ultrasound guided injection

## Abstract

**Background:**

Trigger finger is a common disease with a lifetime prevalence of 2%. One of the frequently preferred non-surgical treatments is blinded injection around the A1 pulley. This study aims to compare the clinical results of ultrasound-guided and blinded corticosteroid injection in the trigger finger.

**Methods:**

In this prospective clinical study, 66 patients who had persistent symptoms of a single trigger finger were included. Patients with similar baseline characteristics such as age, gender, triggering period, and comorbidities were randomized. 34 patients had ultrasound-guided (UG), and 32 had blinded injections (BG). QDASH, VAS, time to return to work, and complications were compared between the groups.

**Results:**

The mean age was 52,66 (29–73) years. There were 18 male and 48 female patients. In the UG, the triggering resolved faster, returning to work was earlier, and the medication period was shorter (*p* < 0.05). A total of 17 patients who had diabetes mellitus received re-injections, 11 of which were in BG and 6 in UG (*p* < 0.05). Although statistically significantly lower scores were obtained in UG at the 1st and 4th weeks in the QDASH and VAS scores (*p* < 0.05), at the 12th and 24 weeks, there was no significant difference (*p* > 0.05).

**Conclusion:**

Using ultrasound guidance for corticosteroid injections is more effective for treating trigger fingers than the blinded method, leading to better results and a faster return to work in the early stages of treatment.

## Background

Trigger finger (TF) is the inflammation and swelling of the retinacular sheath that gradually restricts the mobility of flexor tendons [1]. This sheath forms a roller system intended to maximize the strength and effectiveness of the flexor tendon [2]. The annular pulley (A1) in the volar of the metacarpal head is the most affected in the trigger finger, where the affected finger suffers from pain, clicking, stiffening and immobility [3].TF diagnosis can be easily made with meticulous physical examination and careful examination of the patient history. Findings in physical examination range from sensitivity in the A1 pulley to stuck fingers in flexion [4]. Before resorting to surgery, the initial treatment applied to treat trigger fingers is the conservative treatment regimen encompassing activity modification, non-steroidal anti-inflammatory drugs, joint immobilization, and corticosteroid injections [3]. However, most patients with advanced trigger fingers are treated with release surgery [5]. Since the introduction of corticosteroids in 1953, corticosteroid injection has proven to be an effective treatment, although histopathological examinations of A1 pulleys found no inflammatory component [6]. Therefore, blinded corticosteroid injection is applied around the A1 pulley using the palpation technique and reduces symptoms between 60 and 90% [7, 8]. Upon the widespread access to ultrasonography in recent years, studies have reported the results they obtained focusing on the accuracy of ultrasound (USG)-guided corticosteroid injection [9–11]. USG offers to view the tendon sheath's interior. On the other hand, USG has an extra advantage in showing the potential accompanying pathologies (e.g. ganglion cysts, tendon sheath tumors, tendon sheath effusions, etc.) [12]. There are very few prospective studies comparing the ring ultrasound-guided and blinded injection in the relevant literature. The present study is intended to clinically compare the USG-guided and blinded injection methods to investigate the feasibility of the former approach.

## Materials and methods

The present study was designed as a prospective controlled study and initiated upon the approval of the local ethics committee. The present study included successive patients with Quinnel stage 2,3 or 4 trigger fingers who did not respond to the conservative treatment (splint, non-steroidal anti-inflammatory, drugs and exercise) and gave their consent on the patient consent form. The two groups of patients treated with blinded injection (BG) or USG-guided injection (UG) were compared in terms of demographic data, trigger recovery time, QDASH (Quick Disability of Arm, Shoulder and Hand) scores, VAS (Visual Analogue Scale) scores, time until return to work, complications, and other diseases. QDASH scores of the patients were recorded before the injection and at 1–4 to 12–24 weeks after the injection. Their VAS scores were recorded before and after injection.

Patients over the age of 18 who had no history of treatment or surgery for trigger fingers were included in the study. The exclusion criteria herein were having congenital trigger fingers, having multiple trigger fingers, having a neurological disease, having finger contracture, and having rheumatoid arthritis.

In the blinded injection, patients were positioned in a sitting position with the elbow in 90^o^ flexion, the forearm in supination, and the application area was disinfected twice while applying the palpation technique (on the distal palmar crease and volar side of the metacarpophalangeal joint for the first finger) on the A1 pulley. A mixture of 1 mL of betamethasone sodium phosphate 40 mg/mL and 1 mL of prilocaine hydrochloride 20 mg/mL was used. Fingers were passively stretched and extended before and during the injection to prevent intratendinous application. The syringe was also retracted to ensure the injection was not intravenously applied.

**Fig. 1:**
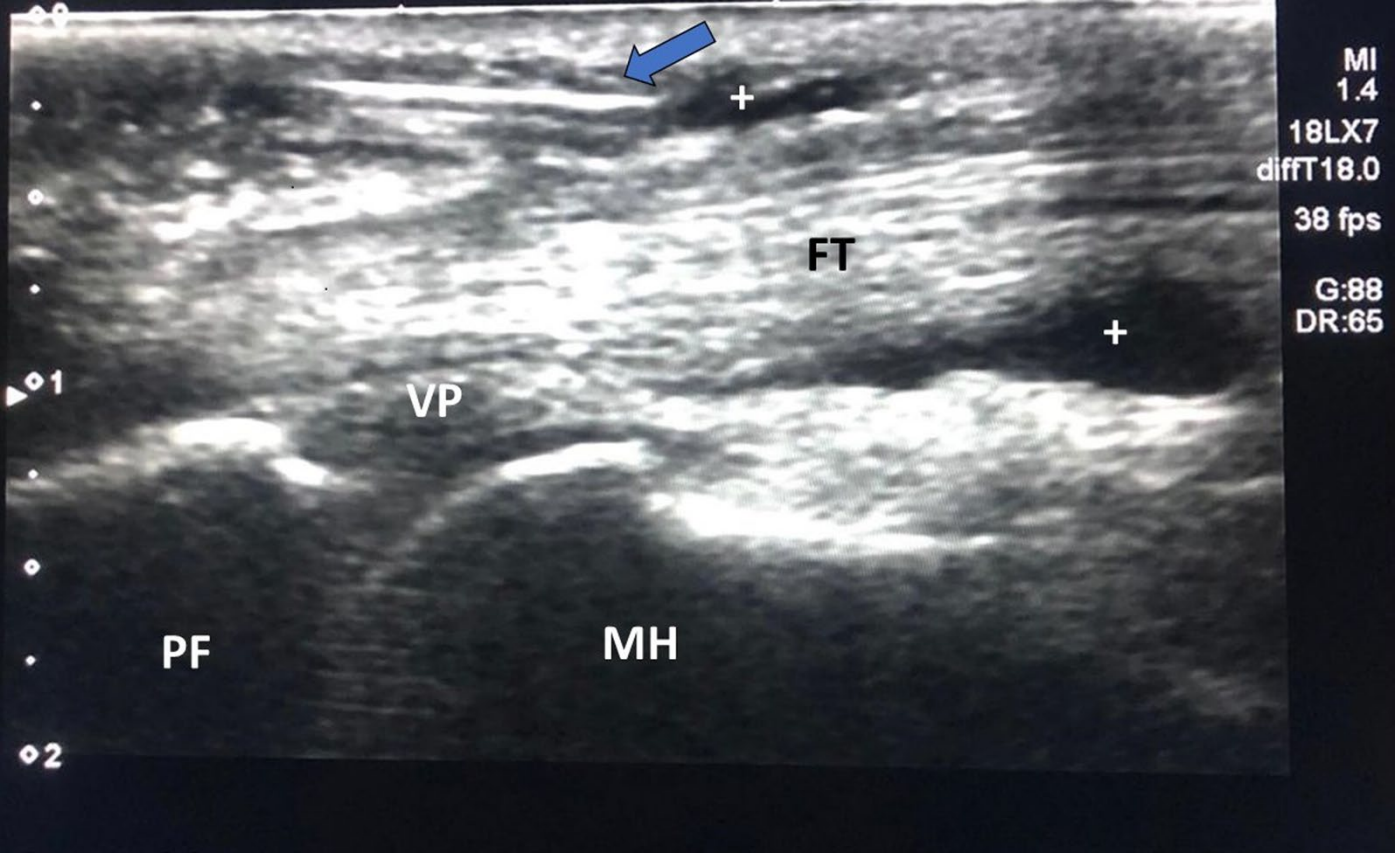
Ultrasound guided trigger finger injection. Arrow: 27 G needle, + : inside of the swollen flexor tendon sheath after injection, *FT* Flexor tendon, *VP* Volar plate, *PF* Proximal phalanx, *MH* Metacarpal head

The Samsung trademark ultrasound device and the LA2-14A linear probe with a frequency of 2.0–14.0 MHz were used for ultrasound-guided injections. In the USG-guided injection, patients were positioned in a sitting position with their elbow in 90^o^ flexion, and their forearm in supination, and the application area was disinfected by putting a silicon support under the hand. A distal-to-proximal approach was used for all UG patients. After ensuring that the USG was positioned in parallel with the flexor tendon, the injection was applied under the A1 pulley in parallel with the tendon and into the tendon sheath using a 5 mL syringe with a 27-gauge needle (Fig. 1).

In both groups of the two techniques, the patients were examined for their VAS and QDASH scores at the time of their first-week follow-up, and in those who demonstrated no improvement, the injection was repeated.

### Statistical analysis

Excel (Microsoft Corporation, WA, USA) was used to collect and manage the study data, and SPSS version 24.0 (IBM Corporation, Armonk, NY, USA) was used to perform data analyses. The quantitative data were presented as mean ± standard deviation, whereas the qualitative data were presented as frequencies with percentages. The Independent t-test was used to detect the differences in normally distributed numerical values. In contrast, the Mann–Whitney *U* test was used to detect the differences in non-normally distributed numerical values. Statistical significance was set at a *p*-value of < 0.05.

## Results

The study included 66 fingers of 66 patients, 18 males and 48 females between October 2020 and May 2021. There were 34 fingers in UG and 32 in BG. The demographics of patients are presented in Table 1.

**Table 1:** Demographic data of patients by groups

	Blinded group (*n* = 34)	Ultrasound group (*n* = 32)	*P*
*Age Mean*			
Female *n* (%)	28 (82,4)	22 (68,8)	*0.255*
Male *n* (%)	6 (17,6)	10 (31,3)	
Right	18 (52,9)	20 (62,5)	
Left	16 (47,1)	12 (37,5)	
*Stage (%)*			
2	26 (76,5)	28 (87,5)	*0.32*
3	6 (17,6)	2 (6,3)	
4	2 (5,9)	2 (6,3)	
*Finger (%)*			
1	21 (61,8)	16 (50)	*0.092*
2	5 (14,8)	0	
3	2 (5,8)	9(28,1)	
4	4 (11,8)	5 (15,6)	
5	2 (5,8)	2 (6,3)	
Hypothyroidism	0	4 (12,5)	*0.006*
Diabetes	16 (47)	14 (44)	
Second dose injection	2 (5,9)	2 (6,3)	*0.95*
Transient Neuropraxy	2 (5,9)	0	*0.493*
Surgery	2 (5,9)	2 (6)	*0.164*

*n*: patient

**Fig. 2:**
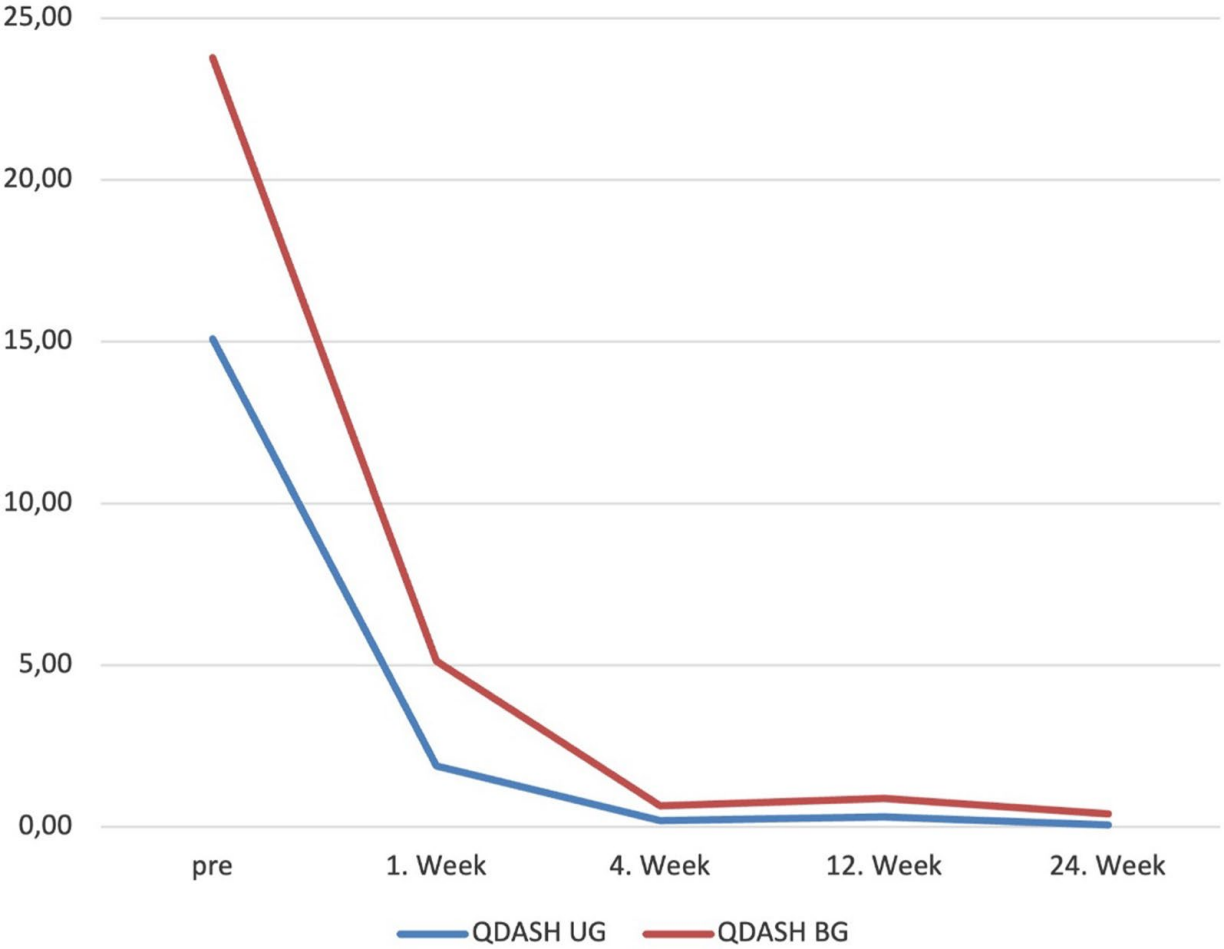
QDASH scores improvements with time by groups. *VAS* Visual analogue scale, *UG* Ultrasound group, *BG* blinded group

**Fig. 3:**
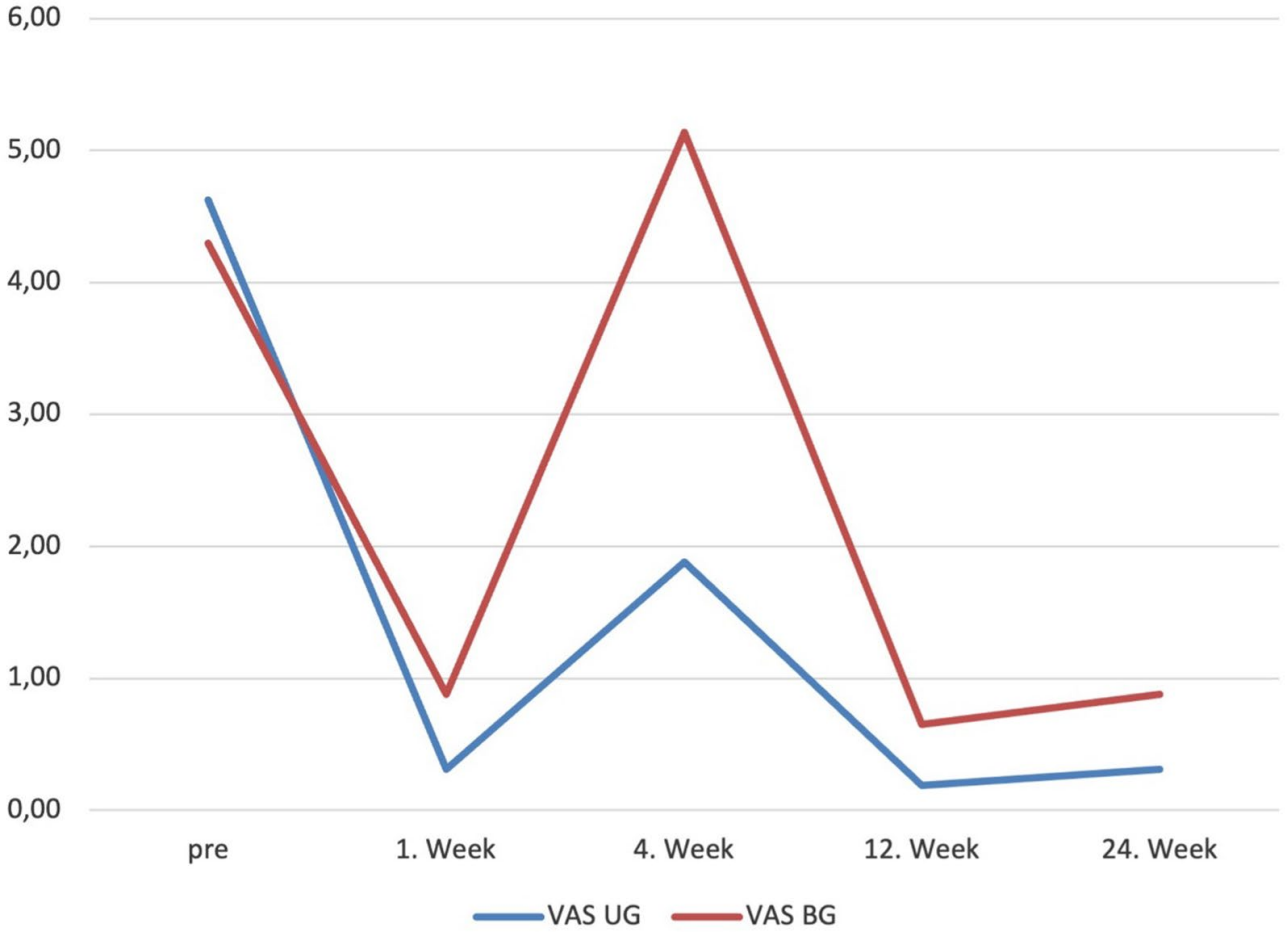
VAS increments by time between groups. *VAS* Visual analogue scale, *SG* Study group, *CG* control group

The mean age was 52,66 (29–73) years. The triggering symptoms were relieved in 59.4% of fingers in the first 3 days, 82.6% in the first week. In addition, 11 patients in BG and 6 in UG required re-injection, and all patients who required re-injection had diabetes mellitus. Patients requiring reinjection in both groups received a second injection in the first week. Thereafter, trigger symptoms were relieved by 91.3% in the second week. Only 4 patients had persistent triggering and pain symptoms after the second injection. 2 patients in BG and 2 patients in UG with persistent symptoms were treated with open surgery. All of them had diabetes mellitus and Quinell stage 3 scores. Patients in the BG required significantly more repeat injections than those in the UG (*p* = 0.000). Two patients in the BG had numbness at the affected finger, which was resolved spontaneously in the second week. 94,2% of patients discontinued NSAIDs after the first week. All patients but two in the blinded group were able to return to work after the 1st week. Between the group comparisons, in the UG, it was found that triggering resolved faster (*p* = 0.000), returning to work was earlier (*p* = 0.004), and medication was shorter (*p* = 0.013). In the QDASH and VAS evaluation, it was observed that statistically significantly lower scores were obtained in the UG at the 1st (*p* = 0.017, *p* = 0.019) and 4th(*p* = 0.005, *p* = 0.009) weeks (Figs. 2 and 3) (Table 2). However, there was no significant difference in VAS and QDASH scores at the 12th (*p* = 0.073, *p* = 0.826) and 24th weeks (*p* = 0.494, *p* = 0.320).

**Table 2:** Comparison between the groups

Variable		Mean	Std. deviation	Std. error mean	P Value*
STAGE	BG	2,11	0,820	0,137	*0.282*
	UG	2,06	0,659	0,115	
RE-INJECTION	BG	0,89	1,008	0,168	***0.000***
	UG	0,30	0,585	0,102	
PRE-QDASH	BG	23,2056	18,62,988	3,10,498	*0.214*
	UG	21,6939	12,08866	2,58,213	
PRE-VAS	BG	4,28	3,239	0,540	*0.172*
	UG	4,39	2,680	0,467	
1st week QDASH	BG	5,1889	7,76,990	1,29,498	***0.017***
	UG	1,8242	3,39,706	0,59,135	
1st week VAS	BG	0,83	1,276	0,213	***0.019***
	UG	0,30	0,770	0,134	
4th week QDASH	BG	0,6167	1,59,257	0,26,543	***0.005***
	UG	0,1818	0,72,692	0,12,654	
4th week VAS	BG	0,39	1,400	0,233	***0,009***
	UG	0,06	0,242	0,042	
12th week QDASH	BG	2,5056	8,40,996	1,40,166	*0.073*
	UG	0,8303	2,78,248	0,48,437	
12th week VAS	BG	0,44	1,027	0,171	*0.826*
	UG	0,42	1,458	0,254	
24th week QDASH	BG	0,7500	1,52,606	0,25,434	*0.494*
	UG	1,0545	1,53,360	0,26,697	
24th week VAS	BG	0,94	1,926	0,321	*0.320*
	UG	1,88	2,459	0,428	

*Independent samples *T*-Test, *p values* lower than *0.05* are considered as statistically significant difference (bold values)

## Discussion

The present study revealed that ultrasound-guided injections in the treatment of trigger fingers achieved faster clinical recovery and an earlier time to return to work compared to the blinded method. The patients who had ultrasound-guided injections demonstrated better recovery at the end of the first 4-week period, particularly regarding VAS scores. After the first month, both groups were found to have similar functional results and VAS values. The most substantial impact of the current prospective controlled study is that considering those who applied ultrasound-guided injection had shorter recovery time, shorter time to return to work and lower rates of repeated injection. The USG-guided injection method was found to be superior in the early period, while the clinical outcomes were the same in both techniques in the long run.

There are many treatment methods available to treat the trigger finger. However, one of the most frequently preferred treatment options is corticosteroid injection [13]. Although the exact action mechanism of corticosteroids is not well-defined in the treatment of trigger fingers, they are thought to act with their anti-inflammatory characteristics. First described by Howard et al., a corticosteroid injection into the tendon sheath offers advantages such as ease of application, applicability in an office setting, low cost, and low rate of complications [14–16]. In addition, ultrasound guidance has become a popular technique to avoid iatrogenic injury when the tendon sheath is located in an anatomically difficult position [9, 10, 17]. However, related literature revealed some studies reporting that out-of-sheath injections also have similar efficacy [18, 19].

Callegari et al. compared open surgery, ultrasound-guided corticosteroid, and hyaluronic acid injection and reported that the ultrasound-guided corticosteroid and hyaluronic acid injection achieved satisfactory outcomes in 14 out of 15 patients (93.3%) [17]. Besides, Bodor and Flossman reported that the ultrasound-guided A1 pulley injection technique is a very effective and minimally invasive treatment option for the trigger fingers, with a 94% success rate within 6 months [10]. The surgical release was applied for all the patients in both groups when the treatment failed. Surgery is more suitable in patients with Quinell stage 4 according to the relevant literature. At this point, we may speculate that associated diabetes, rather than the stage, may play a more critical role in the surgical treatment of stage 3 patients.

In a study where Flensted et al. retrospectively evaluated the recurrence rate after corticosteroid injection, 330 (61%) of the 539 patients had recurrent trigger fingers [20]. The mean recurrence time is 312 days, and the third trigger finger was affected the most commonly. They also argued that this result may be linked to thyroid disease, shoulder diseases, and carpal tunnel syndrome. Similar studies in the literature show that the trigger finger is associated with diabetes, thyroid diseases, carpal tunnel syndrome, and shoulder diseases [15, 21, 22]. As the patients with accompanying upper extremity diseases are excluded from the study, findings concerning associated carpal tunnel syndrome and shoulder diseases were not presented. A notable finding of the present study is that all patients (17) who required repeated injections had associated diabetes mellitus, and 13 (76%) also had associated hypothyroidism.

This study has limitations. The follow-up period is short and the sample size is relatively small. Besides, the USG application has a learning curve and is not available in all orthopedics clinics. The strength of the present study is that it has been well designed as a prospective controlled study including all the follow-ups of the subjects.

The ultrasound-guided corticosteroid injection method was superior to the blinded method in the treatment of the trigger finger due to having a shorter recovery time, an earlier return to work, and fewer injection repetitions. Despite the USG-guided injection method did not differ from the blinded method regarding medium-term clinical outcomes, further prospective studies including larger case series for long-term results are still needed.

## Data Availability

Not applicable.
